# Oligodendroglioma: pathology, molecular mechanisms and markers

**DOI:** 10.1007/s00401-015-1424-1

**Published:** 2015-05-06

**Authors:** Pieter Wesseling, Martin van den Bent, Arie Perry

**Affiliations:** Department of Pathology, VU University Medical Center, De Boelelaan 1117, 1081 HV Amsterdam, The Netherlands; Department of Pathology, Radboud University Medical Center, Nijmegen, The Netherlands; Department of Neuro-Oncology, Erasmus MC Cancer Center, Rotterdam, The Netherlands; Department of Pathology and Neurological Surgery, University of California, San Francisco, CA USA

**Keywords:** Oligodendroglioma, Mixed glioma, Glioblastoma, Histopathology, 1p/19q loss, Molecular marker

## Abstract

For nearly a century, the diagnosis and grading of oligodendrogliomas and oligoastrocytomas has been based on histopathology alone. Roughly 20 years ago, the first glioma-associated molecular signature was found with complete chromosome 1p and 19q codeletion being particularly common in histologically classic oligodendrogliomas. Subsequently, this codeletion appeared to not only carry diagnostic, but also prognostic and predictive information, the latter aspect only recently resolved after carefully constructed clinical trials with very long follow-up times. More recently described biomarkers, including the non-balanced translocation leading to 1p/19q codeletion, promoter hypermethylation of the *MGMT* gene, mutations of the *IDH1* or *IDH2* gene, and mutations of *FUBP1* (on 1p) or *CIC* (on 19q), have greatly enhanced our understanding of oligodendroglioma biology, although their diagnostic, prognostic, and predictive roles are less clear. It has therefore been suggested that complete 1p/19q codeletion be required for the diagnosis of ‘canonical oligodendroglioma’. This transition to an integrated morphological and molecular diagnosis may result in the disappearance of oligoastrocytoma as an entity, but brings new challenges as well. For instance it needs to be sorted out how (histopathological) criteria for grading of ‘canonical oligodendrogliomas’ should be adapted, how pediatric oligodendrogliomas (known to lack codeletions) should be defined, which platforms and cut-off levels should ideally be used for demonstration of particular molecular aberrations, and how the diagnosis of oligodendroglioma should be made in centers/countries where molecular diagnostics is not available. Meanwhile, smart integration of morphological and molecular information will lead to recognition of biologically much more uniform groups within the spectrum of diffuse gliomas and thereby facilitate tailored treatments for individual patients.

## Introduction

For over a century, classification of neoplasms has been based on their microscopic characteristics and the histopathological diagnosis has provided the cornerstone for therapeutic decision-making. The ‘taxonomy’ of tumors of the central nervous system (CNS) in the most recent, i.e., 2007 World Health Organization (WHO) classification of these neoplasms is still largely based on their histopathological features as well [73]. Diffuse gliomas are by far the most frequent neoplasms originating from the CNS parenchyma itself. The common denominator of this heterogeneous group of neoplasms is extensive, infiltrative growth of individual tumor cells and/or groups of these cells (‘diffuse growth’) into the neuropil, i.e., the dense network of neuronal and glial processes of the CNS parenchyma [23].

**Fig. 1:**
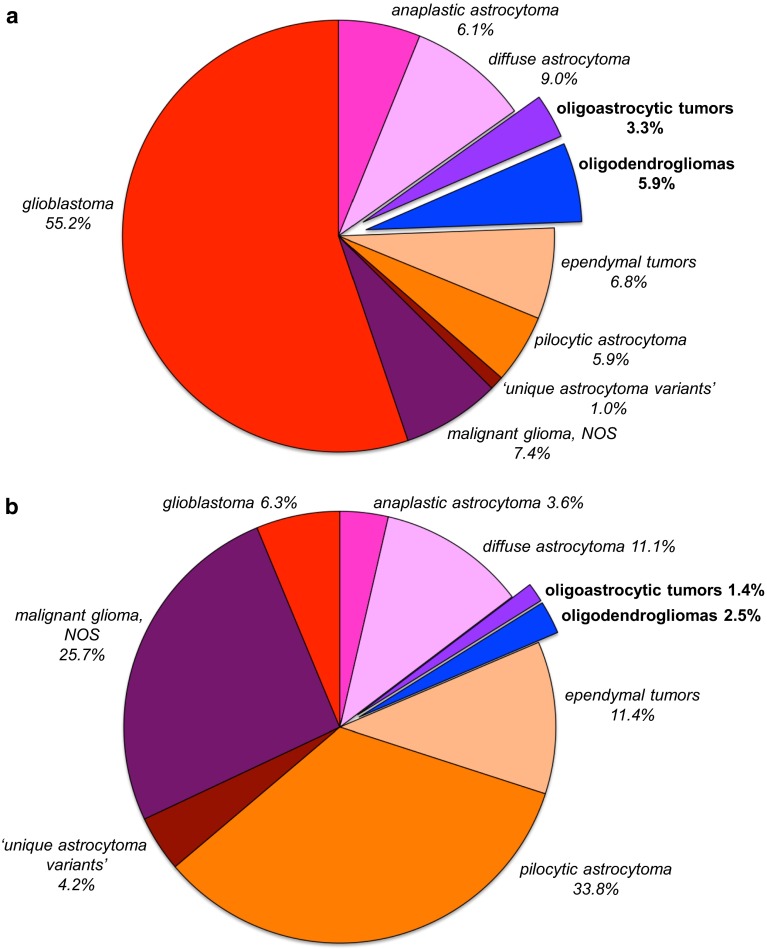
Relative frequency of histopathologically diagnosed oligodendroglial and oligoastrocytic tumors in the spectrum of glial tumors of the CNS: **a** in 95,564 patients of all age groups; **b** in 10,274 children and adolescents (0–19 years). Information extracted from CBTRUS statistical report: NPCR and SEER, 2007–2011 [85]

Over 75 % of the diffuse gliomas in adult patients are astrocytic, about two-thirds of these being represented by the most malignant form, glioblastoma. Oligodendrogliomas and oligoastrocytomas have been generally grouped together as oligodendroglial tumors and account for less than 10 % of the diffuse gliomas (Fig. 1). Of note, in children, glioma variants with a more circumscribed growth pattern are more frequent than diffuse gliomas, and oligodendroglial tumors are rare (<4 % of the primary CNS tumors) [85]. According to the WHO 2007 classification, after typing a diffuse glioma as astrocytic, oligodendroglial, or mixed, these neoplasms are graded as WHO grade II/low grade, WHO grade III/anaplastic or WHO grade IV/glioblastoma (WHO grade I being reserved for more circumscribed gliomas, such as pilocytic astrocytoma). Microscopic features used for grading are the presence/absence of marked mitotic activity, florid microvascular proliferation (MVP, i.e., marked hypertrophy and hyperplasia of endothelial cells and pericytes resulting in a multilayered aspect of the microvessel walls), and necrosis [73]. Unequivocal histological typing and grading of diffuse gliomas is challenging however, especially because the biological diversity of these tumors is difficult to capture in precise microscopic criteria. Also, the samples provided for histopathological analysis are not always (fully) representative. This results in a high rate of interobserver variation in the diagnosis of diffuse gliomas, including oligodendroglial tumors [36, 65, 117].

For the past 20 years, the knowledge on the genetic/molecular underpinnings of gliomagenesis is rapidly expanding. It is now fully clear that certain molecular aberrations carry important diagnostic, prognostic and/or predictive information because they provide clinically relevant information on tumor type, biological behavior, and/or expected response to a particular treatment regimen, respectively. It is therefore to be expected that (like, e.g., sarcomas and hematological malignancies) classification of CNS tumors will increasingly be based on the presence of particular molecular aberrations, and oligodendroglial tumors are amongst the first in line for such a change.

In this review, we reconstruct the transition from a purely morphological to an integrated morphological and molecular scheme for classification of the heterogeneous group of oligodendroglial tumors. After summarizing clinical, radiological and histopathological aspects of these neoplasms (‘Setting the stage’), the molecular underpinnings of oligodendroglial tumors and the diagnostic, prognostic and/or predictive value of these aberrations will be discussed (‘Molecular mechanisms and markers’). In the last part of this article (‘WHO’s next?’), we elaborate on how these markers can be used to reclassify oligodendroglial tumors more objectively, but with some challenges and unanswered questions remaining. For a review on similar aspects concerning diffuse astrocytomas, glioblastomas, and pilocytic astrocytomas the reader is referred to companion articles in the present cluster.

## Setting the stage

Gliomas originate from neural stem cells or glial progenitor cells that develop or maintain glial characteristics [136]. According to the WHO 2007 classification, based on the resemblance of the tumor cells with non-neoplastic glial cells most diffuse gliomas can be typed as astrocytic, oligodendroglial, or mixed (i.e., oligoastrocytoma) [73]. Oligodendroglial tumors most commonly arise in the frontal lobe, somewhat more frequently in males and with the peak incidence in the 5th and 6th decade. Robust enhancement is not a common feature in low-grade examples and suggests transformation to a higher histologic grade [61, 73]. Most oligodendroglioma patients present with seizures. Patients experiencing generalized tonic–clonic seizures were reported to more often have the greatest lesion load in mesial frontal regions, including cortex connected to the genu of the corpus callosum, while in patients with partial seizures, the oligodendrogliomas were located more caudo-laterally in orbitofrontal and temporal lobes but typically sparing cortex connected to the genu, indicating that the genu of the corpus callosum is a major pathway for seizure generalization [130].

Because of their locally aggressive behavior and the fact that they cannot be cured by current therapies, diffuse gliomas are considered one of the most devastating cancers [23]. Progress has been made with oligodendroglioma patients such that current standard of care (surgery, radiotherapy and chemotherapy) yields median survival times of 12–14 years even in WHO grade III examples. Patients with a WHO grade II oligodendroglioma may survive even longer, but sooner or later such tumors generally progress to a high-grade malignant glioma as well. Given the often young age at presentation, however, this patient category still substantially contributes to the high average number of ‘years of life lost’ for patients suffering from a CNS tumor [17]. Rarely, extracranial metastases of oligodendroglial tumors occur (for recent reviews, see [71, 78]). Only a tiny fraction of oligodendroglial tumors is thought to be due to single-gene hereditary syndromes such as biallelic Lynch syndrome [37, 42].

**Fig. 2:**
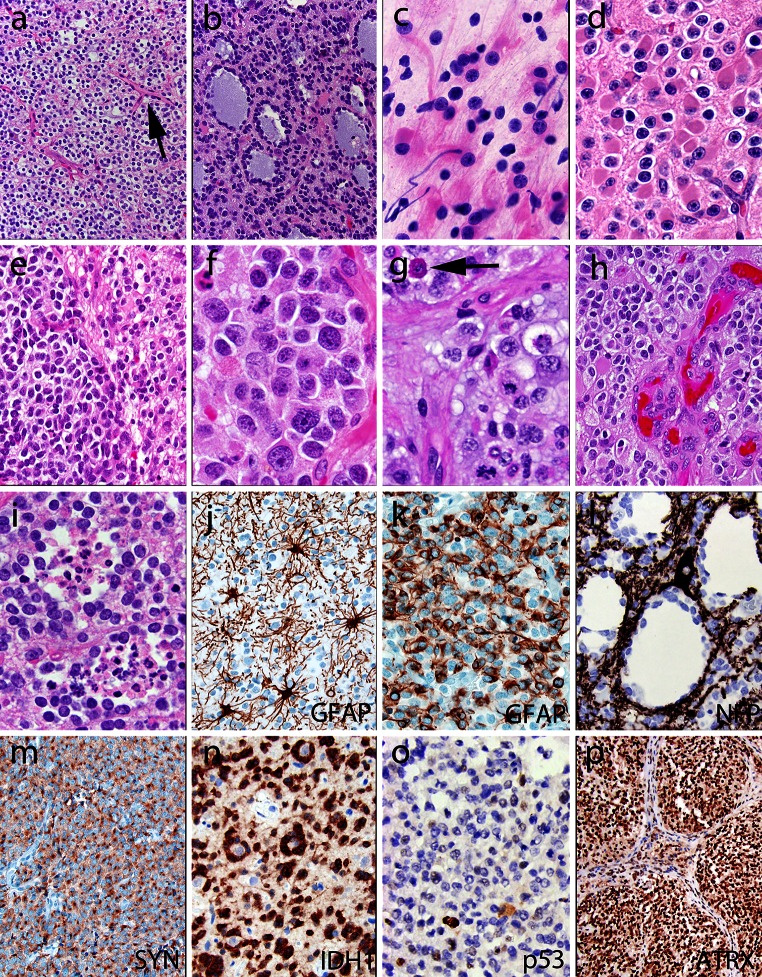
Common histopathologic patterns encountered in classic (IDH mutant, 1p19q codeleted) oligodendrogliomas include: **a** a honeycomb-like arrangement of evenly spaced tumor cells with uniformly rounded nuclei and clear haloes imparting a “fried egg” appearance; additionally, there are branching capillaries (*arrow*) resembling chicken-wire; **b** mucin-filled microcystic spaces; **c**, **d** minigemistocytes with small rounded bellies of eosinophilic cytoplasm and classic nuclear cytology including rounded countours, delicate “salt and pepper” chromatin, sharp nuclear membranes, and small nucleoli (**c** intra-operative smear; **d** tissue section); **e** foci of anaplastic transformation with increased cellularity and enlarged cell size (*left*) in comparison to the low-grade precursor cells (*right*). Anaplastic examples often feature: **f** enlarged epithelioid cells with increased pleomorphism, vesicular chromatin, macronucleoli, but a maintained general sense of nuclear roundness; **g** increased mitotic index and occasional “red crunchy” cells with brightly eosinophilic lysosomal inclusions (*arrow*); **h** microvascular proliferation; and **i** foci of tumor necrosis, more often consisting of collections of apoptotic cells rather than the pattern of pseudopalisading necrosis seen in glioblastomas. Immunohistochemically, some cases are completely GFAP negative, with only reactive astrocytes staining (**j**), while others (**k**) show strong expression in gliofibrillary oligodendrocytes (perinuclear rim and variable tadpole-like tail) or minigemistocytes (not shown). **l** A stain for neurofilament protein often highlights the infiltrative growth pattern by showing entrapped neurons and axons. **m** Tumoral synaptophysin positivity is not uncommon and can include a paranuclear *dot*-*like* pattern. **n** A stain for IDH1-R132H mutant protein highlights perineuronal satellitosis in this positive tumor with cortical involvement. Unlike diffuse astrocytomas, most oligodendrogliomas show only limited p53 immunoreactivity (**o**) and retained ATRX expression (**p**)

As originally described by Bailey and Bucy [7], nuclear features are key for microscopic recognition of an oligodendroglial phenotype, although some architectural growth patterns also provide useful clues (Fig. 2). In low-grade oligodendrogliomas, the nuclei are generally round and uniform with crisp nuclear membranes, delicate chromatin and small-to-inconspicuous nucleoli. In anaplastic examples, cells are frequently enlarged and epithelioid with nuclei that often show increased size and pleomorphism, a more vesicular chromatin pattern and more prominent nucleoli, but the tumor still maintains an overall sense of regularity and nuclear roundness [80, 81]. The clear, perinuclear halo that often is seen in oligodendroglial tumor cells is in fact an artifact of formalin fixation, which is typically absent in frozen sections and rapidly fixed specimens. The combination of the round nuclei and perinuclear haloes results in a fried-egg appearance of individual cells and in a honeycomb architectural pattern of groups of evenly spaced cells.

Oligodendroglial tumors are often highly cellular lesions in central regions with closely packed, relatively small cells. However, in less cellular areas at the tumor periphery, the diffuse infiltrative growth is easily appreciated, often with prominent secondary structure formation, such as clustering of tumor cells around the perikarya of preexistent neurons (satellitosis), under the pial surface (subpial aggregation), and surrounding cortical small vessels (perivascular aggregates). The presence of a branching network of delicate capillaries (chicken-wire pattern) and extensive calcification are common, but not specific additional features in oligodendrogliomas. Rare oligodendroglial tumors show marked pleomorphism (‘polymorphic oligodendroglioma’) or cells arranged in a rhythmic/spongioblastic fashion. Furthermore, part of the tumor cells in oligodendrogliomas may show a gliofibrillary or minigemistocytic phenotype with strong staining for glial fibrillary acidic protein (GFAP) of the cytoplasm, or rarely even signet-ring cell morphology [68, 69, 89]. Typically, oligodendrogliomas in children are histologically similar, but may involve unusual sites such as the posterior fossa and the spinal cord more frequently and lack the molecular background of adult counterparts [102]. Occasionally, a mostly pediatric, slowly progressive oligodendroglial-like proliferation presents as a diffuse leptomeningeal neoplasm [97, 100]. The latter may include the 1p19q codeletion pattern of adult oligodendroglioma, but more frequently harbors 1p deletion without 19q loss [79, 101].

**Fig. 3:**
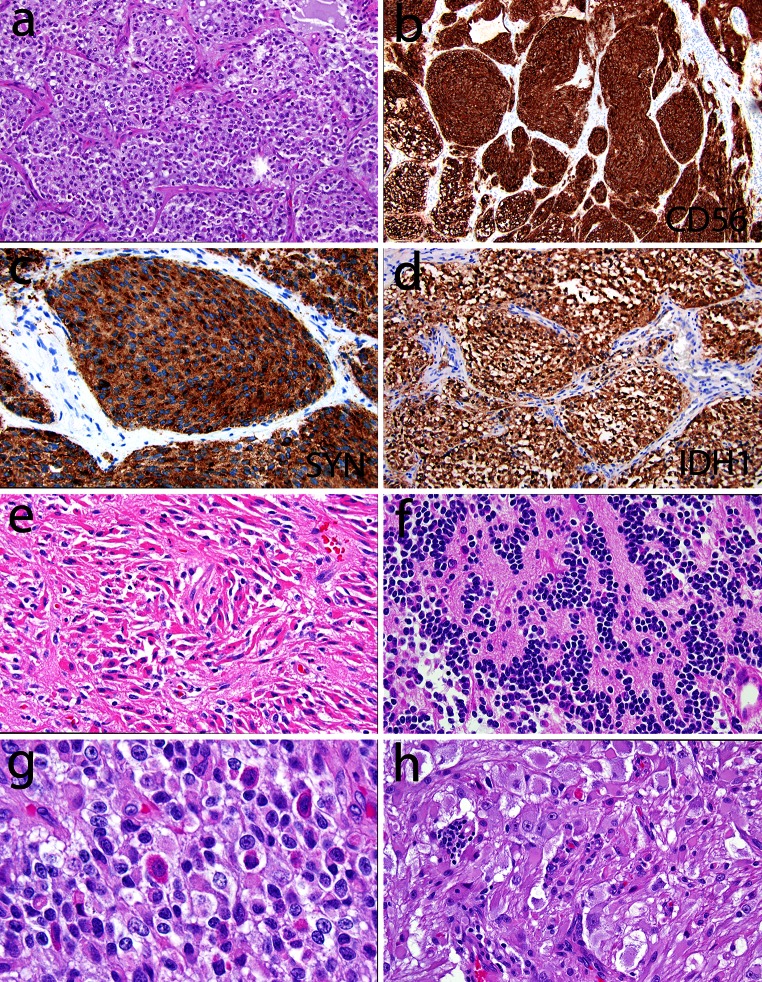
Less common and sometimes diagnostically confusing histopathologic patterns encountered in classic (IDH mutant, 1p19q codeleted) oligodendrogliomas include: **a**–**e** a lobulated or nodular growth pattern can simulate a neuroendocrine tumor, with immunohistochemical pitfalls including CD56 (**b**) and synaptophysin (**c**) positivity; however, demonstration of IDH1 R132H mutant protein (**d**) and 1p19q codeletion confirmed the diagnosis in this case; **e** focal spindled cytology was also encountered, simulating a more astrocytoma-like pattern. Rare examples demonstrate more overt neuronal differentiation, including neurocytic (**f**) and ganglioglioma-like (**g**, **h**). Neurocytic foci often feature slightly smaller and more hyperchromatic nuclei surrounding central collections of delicate pink neuropil (i.e., neurocytic rosettes). In contrast, the case with ganglioglioma-like maturation featured areas of classic anaplastic oligodendroglioma (**g** note also the “red crunchy” cells); FISH studies (not shown) revealed 1p19q codeletions not only in this component, but in neoplastic ganglion cells (**h**) as well

**Fig. 4:**
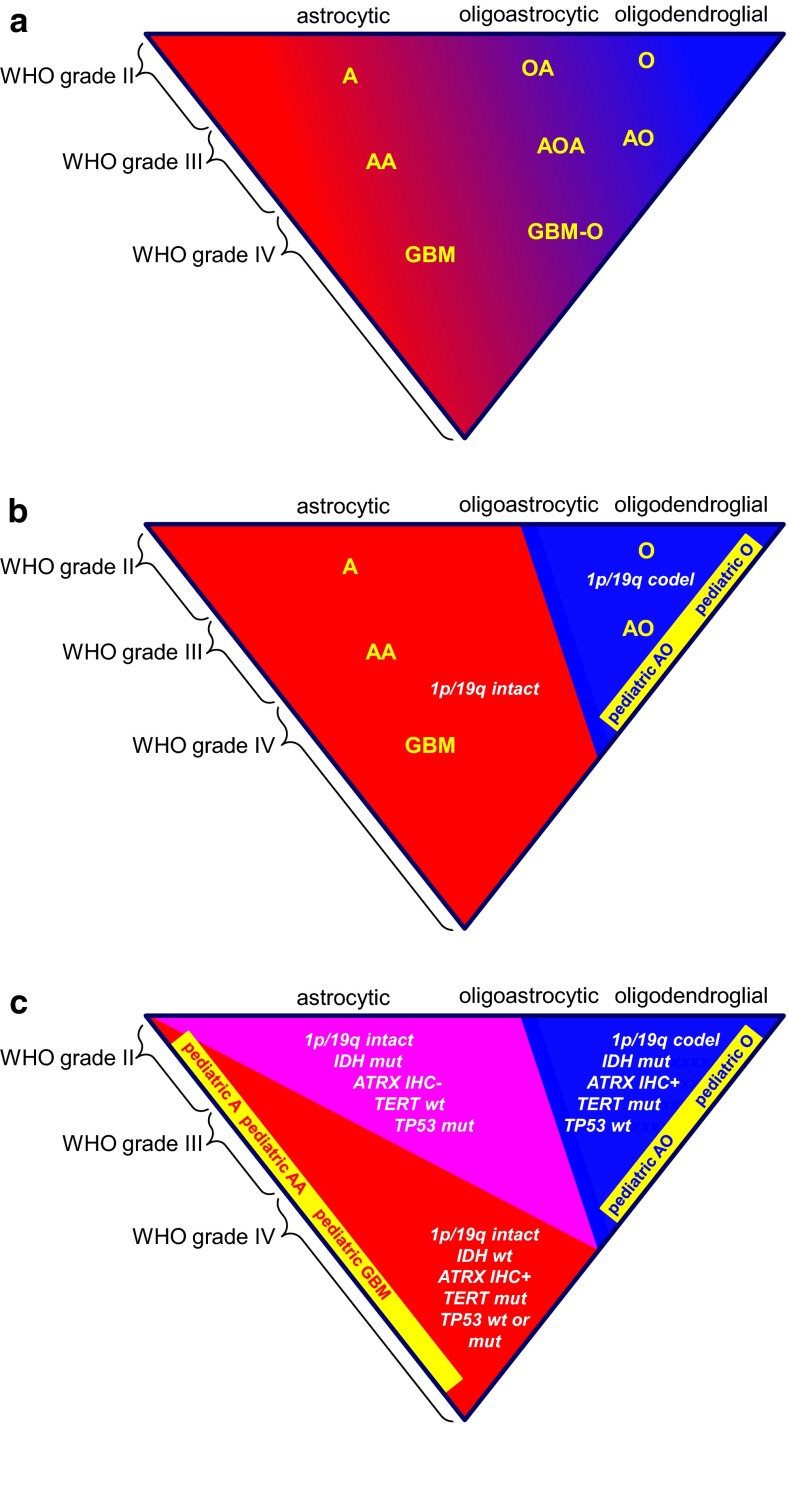
Diffuse gliomas: from histopathologically to molecularly defined entities. Diffuse gliomas histopathologically form a spectrum, both with regard to cell type (astrocytic, oligodendroglial, mixed) and malignancy grade. Especially, delineation of oligoastrocytomas from (more) pure astrocytic and oligodendroglial tumors is poorly reproducible. In practice, (neuro)pathologists who readily accept the existence of mixed gliomas will more liberally diagnose oligoastrocytomas, while those who are skeptical that this entity exists will designate the vast majority of diffuse gliomas as either astrocytic or oligodendroglial (**a**). Introducing 1p/19q codeletion as a defining feature for oligodendrogliomas will in most cases allow for a clear distinction from astrocytic neoplasms and can be expected to drastically reduce the fraction of neoplasms diagnosed as mixed/oligoastrocytic (**b**). Meanwhile, especially in children, CNS tumors resembling classic oligodendrogliomas often lack 1p/19q codeletion; the best position for these ‘pediatric oligodendrogliomas’ in an improved taxonomy of CNS tumors requires further study. **c** Molecular markers helpful in daily clinical practice for state-of-the-art classification of diffuse gliomas. A recently published algorithm proposes immunohistochemistry (IHC) as a first step, allowing for correct classification of esp. the IDH1 R132H mutant protein positive, ATRX negative (and thus ATRX mutated) astrocytomas [96]. Ideally, in other tumors, subsequent molecular testing for 1p/19q codeletion status and/or other IDH1 and IDH2 mutations is performed. Variable testing for other markers can be helpful as well: in IDH mutated (mut) diffuse gliomas demonstration of TERT mutation indicates oligodendroglioma and of TP53 mutation astrocytoma; demonstration of TERT mutation in IDH wild type (wt) diffuse gliomas as well as of EGFR amplification/EGFRvIII strongly indicates high-grade malignant astrocytic tumor/glioblastoma; a KIAA-BRAF fusion gene is typically found in pilocytic astrocytoma, while mutations in the histone genes H3F3A, HIST1H3B/C indicate pediatric type high-grade gliomas (which may also harbor ATRX mutations and show loss of ATRX immunoreactivity). *A*, *O*, *OA* low-grade astrocytoma, oligodendroglioma, oligoastrocytic/mixed glioma, resp.; *AA*, *AO*, *AOA* anaplastic astrocytoma, oligodendroglioma, oligoastrocytic/mixed glioma, resp.; *GBM* glioblastoma, *GBM*-*O* glioblastoma with oligodendroglial component. See also text in this article and the other reviews in this cluster

Histopathological typing of diffuse gliomas is relatively straightforward for tumors at the ‘prototypic ends’. However, there is a surprisingly wide morphological spectrum that includes both common and diagnostically misleading patterns (Figs. 2, 3). As indicated by their name, the histopathological diagnosis of oligoastrocytomas is based on the presence of neoplastic glial cells with morphological characteristics of both astrocytes and oligodendrocytes. These cells may be diffusely mixed or separated, although the latter form is rare [73]. The diagnostic definitions of oligoastrocytomas have been subjective and their diagnosis is poorly reproducible. In fact, conceptual preferences of individual (neuro)pathologists further impact cell type determinations. For instance, those who readily accept the existence of mixed gliomas are likely to more liberally diagnose oligoastrocytomas than those skeptical that this entity exists (Fig. 4a) [16, 35].

While in the past, different systems for the assessment of malignancy grade of oligodendroglial tumors were applied [67], the WHO classification has since gained world-wide acceptance [60, 73]. According to the WHO criteria, an oligodendroglioma without necrosis, florid MVP, or marked mitotic activity is considered as WHO grade II, while the presence of one or more of these features generally warrants an anaplastic designation (WHO grade III). Nevertheless, considerable problems arise in daily practice given the subjectivities of these definitions. For instance, how many mitoses are needed for “marked mitotic activity”? One study reported that finding six or more mitoses per ten high power fields allowed for recognition of oligodendrogliomas with more aggressive (WHO grade III) clinical behavior [36]. Additional challenges arise because the grading criteria for astrocytomas differ and as such, the initial determination of astrocytoma versus oligodendroglioma is often critical to whether the same glioma will be considered low- or high-grade.

In oligodendrogliomas, florid MVP and necrosis do not have the same unfavorable connotation as in diffuse astrocytic neoplasms, and such tumors are still considered WHO grade III. In mixed gliomas, the presence of florid MVP was similarly considered to be consistent with WHO grade III, whereas prior to the 2007 WHO classification, the significance of necrosis was unclear. In a large study published in 2006, necrosis was found to be a statistically significant predictor of poor overall survival for patients with anaplastic oligoastrocytoma, but not for those with anaplastic oligodendroglioma [80]. Others published similar observations [107, 119], leading to the conclusion that for anaplastic oligoastrocytomas, stratification into grade III and IV on the basis of absence or presence of necrosis was justified leading to a ‘glioblastoma with oligodendroglial component’ (GBM-O) category in the WHO 2007 classification [73]. Of note, the concept of GBM-O had already been around for more than a decade [24].

More recently, Appin et al. [4] reported that GBM-Os tend to arise in younger patients, more frequently originate from lower grade precursors and are associated with longer survival. The better prognosis of patients with GBM-O compared to those with classic glioblastoma in this series may then at least partly be explained by the fact that these GBM-Os were more often secondary glioblastomas with *IDH* mutation occurring in younger patients with dedifferentiated tumors [56], a clinical and molecular subtype already known for a better prognosis. Other studies on GBM-O provided conflicting results (see for review [29]). For example, of 339 glioblastoma cases included in the EORTC_26981/NCIC_CE.3 trial and originally diagnosed as glioblastoma by a local pathologist, 15 % was reclassified as GBM-O but this re-grouping lacked prognostic significance [44]. Acknowledging that histopathological distinction of GBM-O from small cell glioblastoma can be difficult, it is likely that the latter variant was overrepresented in some studies taking glioblastomas as a starting point, particularly given their reportedly high frequencies of *EGFR* gene amplification. These data once again illustrate the fact that histopathological discrimination of mixed gliomas from pure counterparts remains subjective and at least partly explain differences in overall survival of patients with anaplastic oligoastrocytomas between various large prospective studies.

Not infrequently, other primary CNS tumors may show oligodendroglioma-like features including fried-egg appearance of (part of) the tumor cells. Examples are glial neoplasms like pilocytic astrocytoma and clear cell ependymoma, as well as neuronal or glioneuronal tumors such as neurocytomas and dysembryoplastic neuroepithelial tumors (DNTs). The presence of Rosenthal fibers and eosinophilic granular bodies in a glial tumor suggests pilocytic astrocytoma, although they can be rarely found in diffuse gliomas as well. Other features that are helpful in this respect include biphasic growth pattern (pilocytic astrocytomas), the formation of true rosettes or perivascular pseudorosettes (ependymal tumors) and the formation of specific glioneuronal elements (DNT). Furthermore, immunohistochemical studies (e.g., dot-like staining for epithelial membrane antigen typically present in ependymal tumors) and occasionally electron microscopy (for ultrastructural demonstration of ependymal or neuronal differentiation) may be helpful in problematic cases. However, synaptophysin staining (for quite some time considered as sound evidence for neuronal rather than glial differentiation) has been described to occur in ‘bona fide’ oligodendrogliomas (and other gliomas) as well, indicating that the progenitor cells from which these tumors are derived are less strictly committed to glial lineage than previously thought [92, 123]. This may also explain that occasionally oligodendrogliomas are reported to display neurocytic or ganglioglioma-like maturation and that differentiation from other, more typical glioneuronal tumors can be challenging [91, 114, 115].

Unequivocal recognition of oligodendrogliomas is hindered by the lack of specific immunohistochemical markers for these tumors [89]. More recently described candidate markers in this respect are Olig2 (a murine bHLH transcription factor expressed in neural progenitors and oligodendroglia and considered to be essential for oligodendrocyte development) and the neuronal intermediate filament alpha internexin (INA). After initial optimism, Olig2 expression was not found helpful for distinguishing oligodendrogliomas from diffuse astrocytomas, nor from DNTs or pilocytic astrocytomas (although lack of Olig2 staining may aid in the diagnosis of neurocytoma or clear cell ependymoma rather than oligodendroglioma) [56, 72, 86, 93, 113]. Similarly, INA expression did not appear to provide the sensitivity and specificity needed for robust recognition of oligodendrogliomas [14, 26, 27]. Very recently, expression of phosphorylated cyclic-AMP responsive element binding protein (p-CREB, a transcription factor involved in gliomagenesis) was described to be present in astrocytomas but largely absent in prototypic oligodendrogliomas [8], but the sensitivity for identifying 1p19q codeleted oligodendrogliomas was only 70 % and the value of this tool for discrimination of diffuse gliomas awaits further elucidation.

While multiple studies show that distinctive histologic features in diffuse gliomas are associated with clinical outcome and with specific molecular alterations, the lack of robust histopathological criteria causes substantial diagnostic interobserver variability, even among experienced neuropathologists [36, 65]. This situation results in undesirable clinical discrepancies [117]. More precise classification of gliomas is urgently needed for adequate assessment of prognosis and appropriate planning of treatment. While improved definitions of morphological features may be helpful in this respect [64], a more promising approach is to integrate molecular data in order to create a more objective and reproducible glioma classification.

## Molecular mechanisms and markers

Like other neoplasms, diffuse gliomas develop as a result of genetic and molecular alterations that further accumulate with tumor progression. These aberrations allow the tumor to acquire assets for sustained survival and growth and to escape normal growth restraining influences [39, 40]. Some of these molecular abnormalities carry important diagnostic, prognostic and/or predictive information, giving support for a major molecular component in future tumor characterization. The most promising markers for oligodendroglial tumors are briefly discussed below.

### Complete 1p/19q codeletion

In 1994, using restriction fragment length polymorphism analysis, Reifenberger et al. [95] reported for the first time that many oligodendroglial tumors show loss of heterozygosity (LOH) for chromosome arms 1p and 19q. Further studies have shown that 1p/19q loss in fact results from a non-balanced translocation *t* (1:19) (q10:p10) with subsequent loss of one of the derivative chromosomes [38, 48]. This explains why it is the loss of the entire arms of 1p and 19q that is related to clinical outcome, rather than smaller terminal or interstitial deletions which occur by a completely different mechanism and are occasionally encountered in astrocytic tumors, such as glioblastoma [47]. Recently, by using genome-wide, 50 base pair single-end sequencing, 1p/19q codeletion was demonstrated to be the only copy number aberration that was stable across spatial regions of low-grade diffuse gliomas and their recurrences [122]. Virtually all 1p/19q codeleted tumors have a pro-neural expression profile, supporting the hypothesis that these gliomas originate from a bi-potential progenitor cell able to give rise to neurons and oligodendrocytes [25].

The high clinical relevance of 1p/19q loss became clear when this marker appeared to be associated with sensitivity to chemotherapy and improved outcome. Cairncross et al. [19] were the first to point out that recurrent anaplastic oligodendrogliomas with 1p/19q codeletion were far more responsive to PCV (procarbazine–ccnu–vincristine) chemotherapy, with virtually all tumors responding. In large prospective randomized studies on diffuse glioma, 1p/19q codeletion was associated with improved overall survival but also with increased benefit of adjuvant PCV chemotherapy given after radiotherapy [18, 118, 127].

Application of ‘strict’ (as opposed to ‘relaxed’) histopathological criteria for recognition of oligodendrogliomas results in a higher correlation with 1p/19q codeleted tumors [2, 51]. In a large series of ‘classic’ glioblastomas, 2–8 % of the primary/de novo and 0–13 % of secondary tumors were reported to show 1p/19q codeletion [83]. In some studies, GBM-Os showed a similar low frequency of 1p/19q codeletion [44], while others reported an increased frequency of codeletions in GBM-Os compared to conventional glioblastoma [4].

### *IHD1*/*IDH2* mutations

Following the initial discovery of isocitrate dehydrogenase (*IDH)* gene mutations in a small subset of especially younger glioblastoma patients with prolonged survival, a high percentage (70–80 %) of *IDH1* and *IDH2* mutations were identified in diffuse grade II and III gliomas, regardless of astrocytic or oligodendroglial cell type. Nearly all mutations involve codon 132 of *IDH1* or the homologous codon 172 of *IDH2*, with c.395G>A (p.R132H) representing >90 % of all *IDH1* mutations [87, 135]. The IDH1 R132H mutant protein can be detected reliably using immunohistochemistry [21]. This will, however, miss approximately 10 % of *IDH* mutations. The *IDH* mutation rate in 1p/19q codeleted tumors approaches 100 % [96, 124]. Further studies have shown that *IDH* mutation is an early event in gliomagenesis (‘driver mutation’), and likely precedes the development of the 1p/19q codeletion, the latter possibly specifically driving a diffuse glioma towards the classic oligodendroglioma morphologic phenotype, whereas superimposed *TP53* and *ATRX* mutations drive it towards the astrocytoma phenotype instead [3, 124, 125].

Through an altered substrate specificity, *IDH* mutations give rise to metabolic alterations, including an increase in production of 2-hydroxyglutarate (2-HG) which can inhibit histone demethylation and induces a glioma hypermethylation phenotype (glioma CpG island methylated phenotype or G-CIMP) [45, 75, 116]. In turn, this leads to a variety of downstream chromatin remodeling and transcriptional alterations. *IDH* status has major prognostic implications for patients with diffuse gliomas, and was suggested to be a better predictive factor for benefit to chemotherapy in the RTOG trial on adjuvant PCV chemotherapy in anaplastic oligodendroglial tumors [20]. *IDH* mutant tumors without 1p/19q codeletion have a worse outcome compared to *IDH* mutant, 1p/19q codeleted grade III tumors, but better than grade II and III tumors without *IDH* mutations.

### *MGMT* promoter methylation and G-CIMP

O6-alkylguanine-DNA-alkyltransferase (AGT), encoded by the O6-methylguanine-DNA-methyltransferase (*MGMT*) gene, is a key repair enzyme that removes alkyl and methyl adducts from DNA, making cells with functional enzyme less sensitive to alkylating and methylating chemotherapy than those incapable of repairing these adducts. Indeed, gliomas with *MGMT* promoter methylation were demonstrated to be more sensitive to the alkylating agent temozolomide (TMZ) [43]. The biomarker value of immunohistochemistry for the repair enzyme itself is less clear [50, 94]. Later on, methylation of very specific regions in the *MGMT* promoter was found to be highly correlated to MGMT expression [6, 77].

Several studies have shown a high rate of *MGMT* promoter methylation in grade II and III gliomas, which subsequently was demonstrated to be related to *IDH* mutational status and G-CIMP. These findings at least partly explain the favorable outcome of *MGMT* methylated tumors after not only chemotherapy or combined chemoradiotherapy, but also after radiotherapy only [120, 127]. Genome-wide methylation studies have shown that *MGMT* promoter methylation is usually part of the G-CIMP phenotype, but about 10 % of G-CIMP positive tumors lack *IDH* mutation [82]. Virtually all 1p/19q codeleted oligodendrogliomas show G-CIMP and *MGMT* promoter methylation. With the Illumina 450 HM beadchip, a specific epigenetic pattern for *IDH* mutated and 1p/19q codeleted tumors can be identified [6, 121, 132]. With combined analysis of *IDH*, *MGMT* and G-CIMP status, it has become clear that the prognostic role of *MGMT* status in grade III tumors is related to *IDH* mutated tumors, whereas *MGMT* methylation status is predictive for benefit to alkylating chemotherapy in the absence of *IDH* mutations in both grade III and IV gliomas (i.e., high-grade astrocytomas) [121, 128, 132]. In EORTC study 26951 on anaplastic oligodendroglial tumors the methylation of specific regions in the *MGMT* promoter was found to be the strongest predictive factor for benefit to PCV chemotherapy [121].

### *CIC* and *FUBP1* mutations

Several other mutations have been identified in 1p/19q codeleted tumors. These include inactivating mutations in the homolog of the Drosophila *capicua (CIC)* and far-upstream binding protein 1 (*FUBP1)* gene, considered to occur secondary to the unbalanced translocation and found to be present in ~50–70 % and 15–30 % of 1p/19q codeleted tumors, respectively. These genes are located on the 19q (*CIC)* and 1p (*FUBP1*) chromosomal arms, supporting their putative roles as tumor suppressor genes [10]. No *CIC* mutations were identified in tumors without 19q loss [103]. The higher rate of *CIC* mutations in pure oligodendrogliomas suggests *CIC* mutations may be related to the phenotypic characteristics of these tumors [10, 54]. *CIC* mutations occur almost invariably with *IDH* mutations, and they are exceedingly rare in other brain tumors. CIC is a downstream component of receptor kinase (RTK) pathways (RTK–RAS–RAF–MAPK) and blocks transcription through binding to a regulatory region. It is negatively regulated by RTK signaling which blocks the function of CIC through MAPK-mediated phosphorylation. This results in the degradation of CIC. *FUBP1* mutations may result in MYC activation or ribosome biogenesis. The additional impact of these alterations on the outcome of patients with 1p/19q codeleted glioma is presently unclear.

### *TERT* and *ATRX*

Two mutually exclusive mutations play a role in telomere maintenance in gliomas: telomerase reverse transcriptase (*TERT*) mutations in hot spot promoter regions C228T, C250T result in increased expression of telomerase. Paradoxically, these mutations are present in both 1p/19q codeleted oligodendrogliomas and in *IDH* wild type high-grade gliomas [5, 59]. Virtually all 1p/19q codeleted tumors carry *TERT* mutations. Gliomas with *TERT* mutations in the presence of 1p/19q codeletion (i.e., oligodendrogliomas), have a very favorable outcome where the same *TERT* mutations in the absence of this codeletion or *IDH* mutations herald a grim prognosis (comparable to glioblastoma) [70]. *TERT* mutations are mutually exclusive with *ATRX* (alpha thalassemia mental retardation syndrome X linked) mutations, which are instead seen in the majority of WHO grade II-III astrocytomas and secondary glioblastomas. Critical for normal telomere homeostasis, these mutations are associated with the alternative lengthening of telomeres (ALT) phenotype, *IDH* and *TP53* mutations, but not with 1p/19q loss [58, 62, 96, 104, 131]. Most diffuse gliomas thus show either *ATRX* or *TERT* mutations. The mechanisms involved in escaping senescence in the remaining set of gliomas still await further elucidation.

### *EGFR* and chromosome 7

*EGFR* amplifications are virtually mutually exclusive with 1p/19q codeletion and with *IDH* mutations [52]. This suggests that these tumors follow a different oncogenic route from the start. The presence of polysomy of chromosome 7 and amplification of the epidermal growth factor receptor (*EGFR*) gene is associated with *TERT* mutations and a prognosis similar to primary glioblastoma in general. The presence of *EGFR* amplification in histologically pure anaplastic oligodendrogliomas is indicative of glioblastoma [31]. The small cell variant of glioblastoma has morphological similarities to anaplastic oligodendroglioma but carries *EGFR* amplification in 70 % of cases [90].

### Anaplastic oligodendrogliomas

Anaplastic oligodendroglial tumors usually have additional genetic aberrations, in particular 9p LOH and/or deletion of the *CDKN2A* gene (p16), *PIK3CA* mutations, and polysomies [10, 11, 13, 31]. Recent data of The Cancer Genome Atlas (TCGA) consortium on lower grade (WHO grade II and III) gliomas show that *IDH* mutations in these gliomas are virtually mutually exclusive with homozygous deletion of *CDKN2A* and with amplification of *EGFR* [1, 12]. As discussed above, *EGFR* amplification mainly occurs in 1p/19q intact, *IDH* wild type gliomas and indicates the diagnosis glioblastoma. Some studies suggested shortened recurrence free survival times or poorer prognosis for oligodendroglial tumors with polysomy of 1p and 19q, but the exact importance of this finding is not yet clear [108, 129].

### Pediatric oligodendroglial tumors

Pediatric and some young adulthood oligodendrogliomas are different from oligodendrogliomas in adults in that they show neither *IDH* mutations nor combined 1p/19q codeletion and thus represent a different disease [21, 63, 102, 111]. The rare childhood examples with *IDH* mutations and 1p19q codeletions almost always occur in adolescents, suggesting that they indeed carry the adult type of oligodendroglioma. In a similar vein, some young adult patients with tumors resembling oligodendroglioma, but lacking *IDH* mutations and 1p/19q codeletion may harbor the pediatric type of oligodendroglioma. This is similar to many other pediatric neoplasms that resemble an adult counterpart, but differ genetically and biologically [34, 110]. Given that it is difficult to define entities by a lack of findings, an objective definition for pediatric oligodendroglioma will likely remain a challenge until these tumors are better characterized at the molecular level. Recently, disseminated oligodendroglioma-like leptomeningeal neoplasms (an entity that predominantly affects children) were reported to frequently harbor concurrent BRAF-KIAA1549 gene fusions and 1p deletion, generally without 19q deletion [101].

### Intratumoral genetic heterogeneity

In a recent study sequencing the exomes of 23 low-grade gliomas and their recurrences, in 43 % of cases, at least half of the mutations in the initial tumor were undetected at recurrence, including mutations considered to be driver mutations such as *TP53* and *ATRX*. These findings suggest that recurrent tumors are often seeded by cells derived from the initial tumor at a very early stage of their evolution [55]. Indeed, other groups reported substantial intratumoral genetic heterogeneity (ITGH) in diffuse gliomas [88, 109, 122]. Adequate understanding of the consequences of such ITGH for the diagnosis and treatment of these tumors needs further study. Moreover, particular (chemo) therapeutic strategies/compounds may elicit certain molecular aberrations in tumor cells. In the study of Johnson et al. [55], tumors from 6 of 10 patients treated with the temozolomide (TMZ) were hypermutated and harbored driver mutations in the RB (retinoblastoma) and Akt-mTOR (mammalian target of rapamycin) pathways, thereby bearing the signature of TMZ-induced mutagenesis.

## Who’s next?

The distinct differences in outcome of the various molecularly defined subsets even within morphologically similar types and grades of diffuse gliomas summarized above is now ready to be translated into a refined, more objective glioma classification with associated clinical, prognostic, and predictive implications. In fact, schemes were already proposed for a classification that is based on assessment of molecular aberrations rather than purely on histopathology [126]. Based on the available data, ‘canonical oligodendroglioma’ in adult patients could be defined as a diffuse glioma with complete 1p/19q codeletion, most of which will also have an *IDH* and a *TERT* mutation (Fig. 4b). Indeed, these tumors have the best outcome in the group of diffuse gliomas and show increased sensitivity to adjuvant PCV chemotherapy. Of note, 1p/19q status is most likely not the ultimate identifier of sensitivity to adjuvant (PCV) chemotherapy, better candidate markers being *IDH* mutation, G-CIMP and *MGMT* promoter methylation.

The question then arises how to call tumors with clear discrepancy between morphology and molecular information. Diffuse gliomas with intact 1p/19q may have oligodendroglial phenotype, but have a less favorable outcome especially if no *IDH* mutation is present. Vice versa, in many larger series on glioma a limited number of tumors diagnosed as low grade or anaplastic astrocytoma (5–10 %) has combined 1p/19q loss, most of these are *IDH* mutated. Several studies have now demonstrated that the molecular classification corresponds better with outcome than histopathology.

Recently, a group of neuropathologists with ample expertise in histopathological and molecular diagnosis of CNS tumors reached consensus and formulated the International Society of Neuropathology-Haarlem consensus guidelines (ISN-Haarlem guidelines) on how molecular information could be incorporated into an updated version of the WHO classification. Salient recommendations of this group included that: (1) diagnostic entities should be defined as narrowly as possible to optimize interobserver reproducibility, clinicopathological predictions and therapeutic planning; (2) diagnoses should be ‘layered’ with histologic classification, WHO grade, and molecular information listed below an ‘integrated diagnosis’ (Table 1); (3) determinations should be made for each tumor entity as to whether molecular information is required, suggested or not needed for its definition; (4) some pediatric entities should be separated from their adult counterparts [74]. The layered diagnostic format allows to leave the ‘molecular layer’ empty and to state in the integrated diagnosis that molecular testing was not performed. However, with the present status of molecular knowledge it is clear that molecular information is required for optimal diagnosis of diffuse gliomas and that in this context for most cases an ‘empty molecular layer’ must be viewed as shortcoming.

**Table 1:** Layered format for integrated morphological and molecular diagnosis of CNS tumors as proposed in ISN-Haarlem guidelines [74]

**Layer 1, integrated diagnosis:** integrating all ‘highlights’ from tissue-based information as described in layers 2–4; until molecular information becomes available, this can be filled out initially as ‘pending’
*Example 1: anaplastic oligodendroglioma (WHO grade III); 1p/19q codeleted*
*Example 2: low-grade astrocytoma (WHO grade II); IDH-mut, ATRX loss*
**Layer 2, histological classification:** standard microscopic diagnosis
*Example 1: glioblastoma with oligodendroglial component*
*Example 2: low-grade mixed glioma*
**Layer 3, WHO grade:** reflecting natural history; may be left out when uncertain or otherwise confusing
*Example 1: WHO grade III or IV (dependent on molecular information)*
*Example 2: WHO grade II*
**Layer 4, molecular information:** synoptic account of type of test(s) performed and the results obtained; may temporarily be filled with ‘pending’ as well or with ‘not done’ (preferably with a reason, e.g., ‘not considered necessary’, ‘not available’, ‘insufficient tissue for testing’)
*Example 1: IDH1-R132H + (IHC), PTEN deletion (FISH), 1p/19q codeletion (FISH)*
* Example 2: IDH1-R132H + (IHC), p53 ++ (IHC), ATRX − (IHC)*

**Fig. 5:**
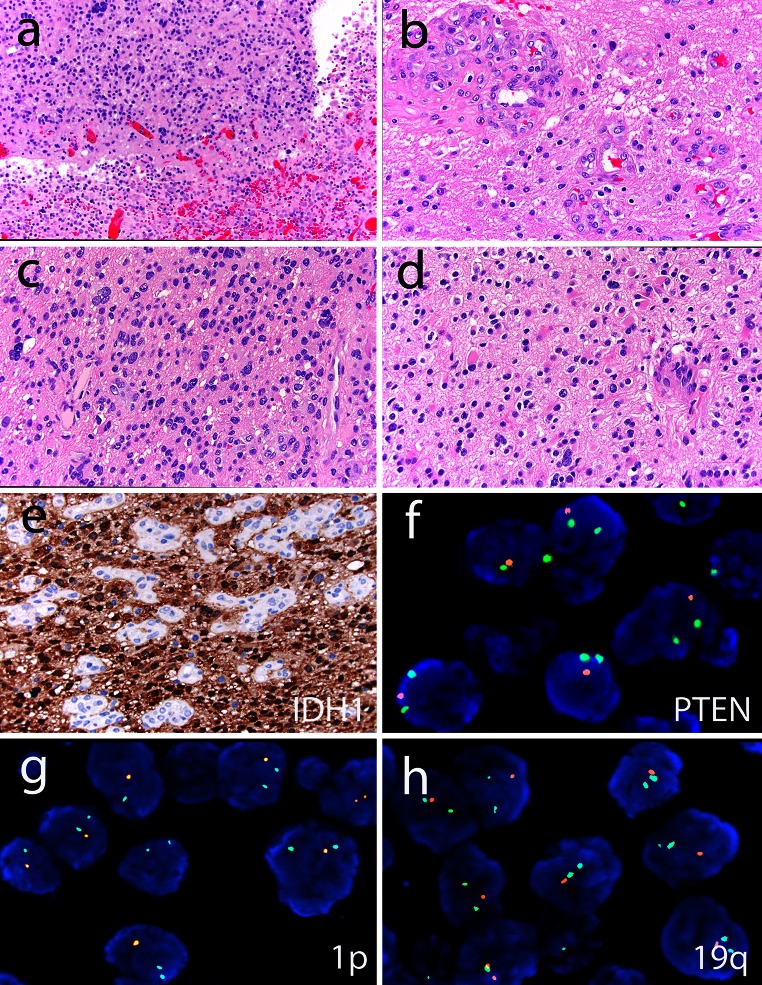
Example of how ‘molecular reclassification’ may affect tumor grade. This tumor, previously diagnosed as GBM-O, WHO grade IV featured mostly cells resembling astrocytoma (**a**, **c**) and included necrosis (**a**) and microvascular proliferation (**b**). Cells resembling oligodendroglioma were seen only focally (**d**). The expression of IDH1-R132H mutant protein suggested that this represents either a secondary glioblastoma or an anaplastic oligodendroglioma, whereas the PTEN deletion identified by FISH is more common in glioblastoma (**f**; centromere 10 probe in *green* and PTEN probe in *orange*). Given the combined deletions of chromosome 1p (**g**; 1p probe in *orange* and 1q probe in *green*) and 19q (19p probe in *green* and 19q probe in *orange*), however, this case would be reclassified as an anaplastic oligodendroglioma, WHO grade III based on the most current recommendations

**Fig. 6:**
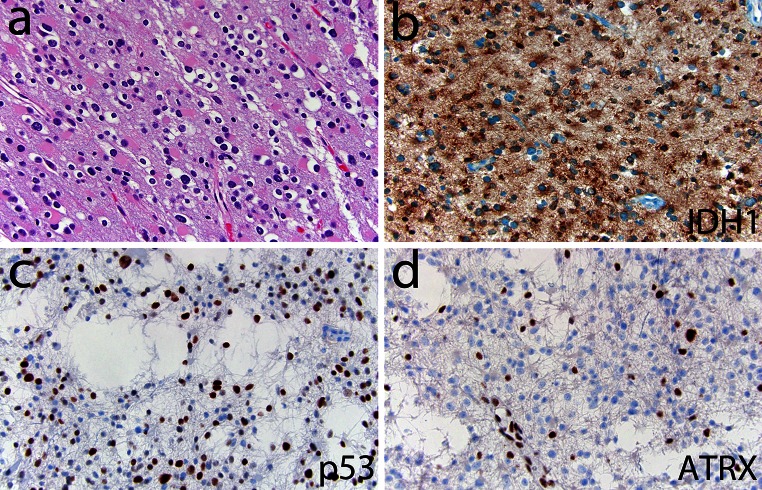
Example where ‘molecular reclassification’ does not affect tumor grade. This previously diagnosed oligoastrocytoma, WHO grade II (**a**) with IDH1 R132H mutant protein (**b**) would now be reclassified as a diffuse astrocytoma, WHO grade II based on strong p53 expression (**c** correlating well albeit imperfectly with TP53 mutation) and loss of ATRX expression (**d** note positive expression in non-neoplastic nuclei serving as a positive internal control)

Example 1 and 2 are further illustrated in Figs. 5 and 6, respectively

*IHC* immunohistochemistry, *FISH* fluorescent in situ hybridization

For molecular subtyping of diffuse gliomas several markers may be exploited (Fig. 4c). Elaborating on the suggestions in the ISN-Haarlem guidelines, a recent study on adult diffuse low- and high-grade gliomas demonstrated that the integrated morphological and molecular diagnosis (incorporating information on *ATRX*, *IDH* and 1p/19q codeletion) had significantly greater prognostic power for overall and progression-free survival than the original, histopathological diagnosis. Acknowledging that *ATRX* mutations in *IDH* mutant diffuse gliomas almost never co-occur with 1p/19q codeletions and result in loss of immunohistochemical ATRX staining of tumor cell nuclei, an algorithm was proposed for a stepwise diagnostic approach that could reduce the number of molecular analyses needed for a final diagnosis: initial immunohistochemistry for ATRX and IDH1 R132H mutant protein, when necessary (e.g., in case of positive ATRX staining of tumor cell nuclei and negative staining for IDH1 R132H) followed by 1p/19q analysis and subsequently by *IDH* sequencing [96]. Alternatively, assessment of *TERT* and/or *TP53* mutations may be exploited for establishing such a prognostic classification, canonical oligodendrogliomas typically being *TERT* mutated and *TP53* wild type (Fig. 4c). Immunohistochemistry for p53 protein (strong staining of a large percentage of tumor cell nuclei) has been reported as a highly specific, but moderately sensitive surrogate marker for the presence of a *TP53* mutation in gliomas [112].

Several studies have now revealed that molecular and epigenetic/methylation characteristics allow reclassification of oligoastrocytomas or morphologically ambiguous diffuse gliomas into either oligodendroglioma or astrocytoma. Based on these conclusions, some authors suggest that it is time to say farewell to oligoastrocytoma as an entity [104, 133]. Indeed, the diagnosis of mixed gliomas has suffered amongst the greatest levels of discordance, even among expert neuropathologists. Already in 1997 the finding that low-grade oligoastrocytomas generally have either 1p/19q codeletion or *TP53* mutation made the authors question the presence of truly mixed gliomas [76]. GBM-O is in fact nothing more than the grade IV variant of oligoastrocytoma and all of the caveats discussed above pertain. Distinct molecular subsets can easily be carved out of the GBM-O category and placed into more clinically uniform subtypes, such as primary/*IDH* wild type glioblastomas, secondary/*IDH* mutated glioblastomas, and 1p/19q codeleted anaplastic oligodendrogliomas. With the adoption of an integrated morphological and molecular diagnosis from a clinical perspective, the need for a separate mixed diffuse glioma (incl. GBM-O) category will largely disappear [32, 41, 53, 83].

The suggestion that it is time to say farewell to oligoastrocytoma as a diagnosis immediately elicited case reports of tumors showing molecular features of composite oligodendroglioma and astrocytoma [46, 134]. Evidently, while molecular classification allows for more stringent definition of the vast majority of diffuse gliomas, there will always be single cases that do not readily fit into the scheme. Further investigations are required to sort out if such ‘outliers’ justify the creation of separate diagnostic categories [105].

Introducing a new, integrated morphological and molecular definition of subgroups of diffuse gliomas will in itself already lead to a change in WHO grade in some cases (see Figs. 5, 6 for examples). Also, evaluation of the exact criteria to be used for grading and the significance of such grading within molecular subgroups is warranted [33]. A recent study of 558 WHO grade II and III diffuse gliomas revealed that mitotic index was significantly associated with outcome in *IDH* wild type but not in *IDH* mutant tumors [84].

**Fig. 7:**
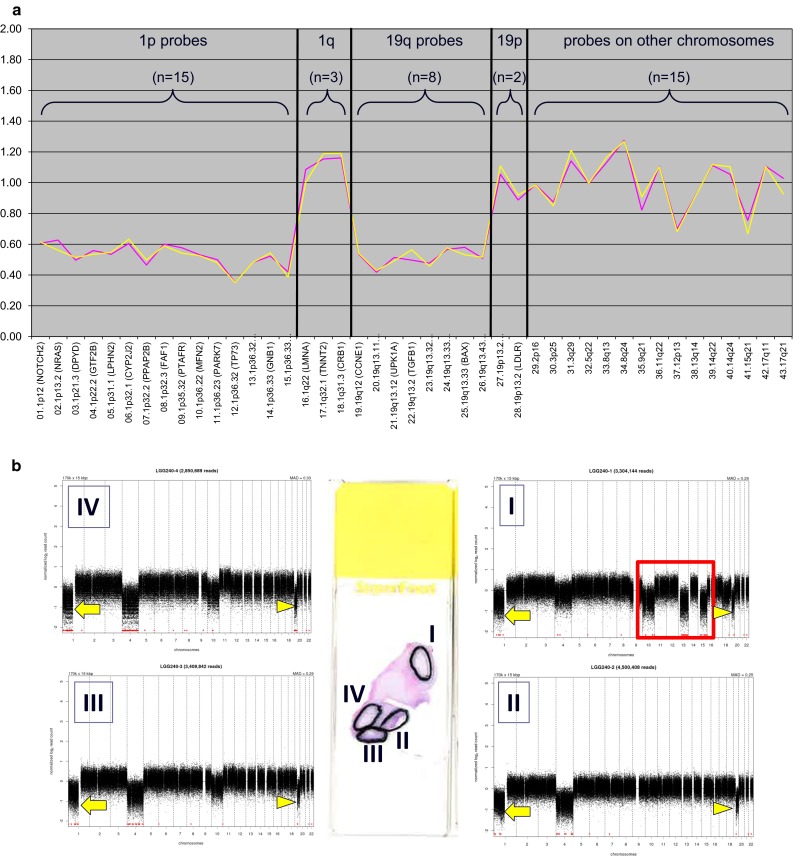
For unequivocal assessment of 1p/19q codeletion in oligodendroglial tumors, detection of whole-arm losses is key. In order to avoid detection of false-positive cases with partial 1p and/or 19q loss, ideally a test is used that allows for analysis of multiple loci along each chromosome arm. **a** Example of an oligodendroglioma of which formalin-fixed, paraffin-embedded tissue was analyzed for 1p/19q status by multiplex ligation-dependent probe amplification (MLPA). With this platform, probes for 15 loci on chromosome 1p and 8 loci on 19q are used to interrogate these chromosome arms for loss or gain [the rest of the probes (3 on 1q, 2 on 19p, 15 on other chromosomes) serve as copy number references]. The test is performed in duplicate (hence the *two lines*) and demonstrates a clear loss for all 1p and 19q probes in this case (see [49] for technical details). **b** Massively parallel sequencing (MPS) allows for much more detailed analysis of copy number aberrations in the (tumor) genome. This is an example of a low-grade oligodendroglioma of which four different regions were separately analyzed. Interestingly, while complete losses of chromosome arms 1p (indicated by the *arrow*) and 19q (*arrowhead*) were uniformly present in the different regions, other copy number aberrations were only detected in some regions (most strikingly in area I, see region marked by *red box*). As the deletions for 1p and 19q loss in the different regions are at a similar level, this heterogeneity for copy number aberrations of other chromosomes cannot simply be explained by a difference in percentage of tumor cells in the different areas and must be interpreted as intratumoral genetic heterogeneity. Image **b** is modified from [122]

Other challenges that need to be tackled are how to put pediatric oligodendrogliomas (known to lack 1p/19q codeletions) in the taxonomy of oligodendroglial tumors and which platforms and cut-off levels are the most ideal to be used for demonstration of particular molecular aberrations. The different platforms for detection of the relevant markers have their own strengths and weaknesses [45, 98]. Importantly, for recognition of the clinically relevant 1p/19q codeletion, assays capable of demonstrating whole-arm losses are preferable, in order to minimize the risk of false positives from incomplete 1p and 19q losses (Fig. 7).

In the more distant future, the therapeutic management of individual patients may be determined by their ‘cancer signature’ rather than by the traditional pathological diagnosis [22]. For instance, the presence of an *IDH* mutation may provide an opportunity to apply specific, IDH targeted treatment, no matter if the diagnosis was astrocytoma or (1p/19q codeleted) oligodendroglioma [15, 106]. Furthermore, now that diffuse gliomas are increasingly diagnosed based on presence or absence of specific molecular aberrations, one can imagine that some of these tumors may be diagnosed by non- or minimally invasive approaches. Several groups already reported that molecular subgroups of gliomas (e.g., *IDH* mutant gliomas) may be recognized as such by advanced magnetic resonance evaluation [28, 30, 57]. Alternatively, ‘liquid biopsies’ may increasingly be used to detect particular markers in blood of glioma patients [9, 66]. It remains to be seen though if such non-invasive methods will ultimately provide information that is detailed and robust enough to replace tissue-based morphological and molecular analysis. Moreover, since the benefits of near-total resection of diffuse gliomas have long been recognized, tissue may continue to be obtained in most cases even if a diagnosis may be made by other means.

In conclusion, after almost a century of a histopathologically based classification of CNS tumors, we are now experiencing the transition towards an integrated morphological and molecular definition of diffuse gliomas, with in some situations the clinical relevance of the molecular findings overriding the relevance of the histopathological diagnosis. This integrated diagnostic approach allows for much more robust recognition of biologically different subgroups within the spectrum of diffuse gliomas, thereby facilitating tailored treatment for individual patients. The official updating process of the 4th edition of the WHO classification of CNS tumors has just begun and ‘the jury is still out’ with regard to what exactly the definitions for subgroups of diffuse gliomas will look like.

It can be expected that in the updated WHO classification (publication of which is scheduled early in 2016) demonstration of (complete) 1p/19q codeletion will indeed be required for the diagnosis of ‘canonical oligodendroglioma’ and that the (poorly reproducible) diagnosis of mixed gliomas will largely disappear. Such a change will bring new challenges as well, e.g., regarding how criteria for grading within the molecular groups should be adapted, how pediatric oligodendrogliomas should be defined, which platforms and cut-off levels should ideally be used for molecular diagnostics, and how to stay connected with centers/countries where molecular testing is not available. Also, acknowledging that our knowledge on the molecular underpinnings of cancer will continue to rapidly expand, it may well be necessary to prepare for more frequent updates of tumor classifications than we have seen so far. A multidisciplinary team-effort is needed to do derive the right balance between state-of-the-art science and clinical practicality in this respect [74, 99].
